# The unrecognised cost of cancer patients' unrelieved symptoms:a nationwide follow-up of their surviving partners

**DOI:** 10.1038/sj.bjc.6600271

**Published:** 2002-05-03

**Authors:** U Valdimarsdóttir, ÁR Helgason, C-J Fürst, J Adolfsson, G Steineck

**Affiliations:** Department of Clinical Cancer Epidemiology, Inst. Oncology-Pathology, Karolinska Institute, Box 4402, 10268 Stockholm, Sweden; The Oncological Centre, M:08, Karolinska Hospital, 17176 Stockholm, Sweden; Stockholms Sjukhem Foundation, Mariebergsgatan 22, 11235 Stockholm, Sweden; Stockholm Centre for Public Health, Box 17533, 11891, Stockholm, Sweden

**Keywords:** terminal care, symptom control, family, bereavement, adaptation, psychological

## Abstract

We investigated if a cancer patient's unrelieved symptoms during the last 3 months of life increase the risk of long-term psychological morbidity of the surviving partner. All women (*n*=506) living in Sweden under 80 years of age, who lost their husband/partner owing to cancer of the prostate in 1996 or of the urinary bladder in 1995 or 1996 were asked to answer an anonymous postal questionnaire, 2–4 years after their loss. The widows' psychological morbidity was associated with the patient's unrelieved mental symptoms. When the patient was perceived to have been very anxious during last three months of life (compared to no observed symptoms) the relative risks for the widows' psychological morbidity were: 2.5 (1.4–4.3) for depression and 3.4 (1.4–8.2) for anxiety. When comparing reports of the patient's pain (much *vs* no), the relative risks were 0.8 (0.5–1.2) for widowhood depression, and 0.8 (0.4–1.7) for widowhood anxiety. The patients were found to have had adequate access to physical pain control but poor access to psychological symptom control. Efficiency in diagnosing and treating psychological complications of terminally ill cancer patients may not only improve their quality of life but possibly also prevent long-term psychological morbidity of their surviving partners.

*British Journal of Cancer* (2002) **86**, 1540–1545. DOI: 10.1038/sj/bjc/6600271 www.bjcancer.com

© 2002 Cancer Research UK

## 

Efficient symptom control (physical and psychological) during a cancer patient's last months of life is undoubtedly a primary goal of palliative care. The delay or failure to provide symptom control results in painful experiences for the patients and their significant others. Moreover, studies have shown that the well-being of patient and partner is interrelated (Cassileth *et al*, 1985; Kurz *et al*, 1995; Kristjanson *et al*, 1996; Hodgson *et al*, 1997). However, it is not known whether the patient's unrelieved symptoms result in the permanent harm of long-term psychological complications of the surviving partner.

Evidently the loss of a loved life companion from chronic illness such as cancer induces sorrow that diminishes over time but may persist for a long time. On top of this, the conditions before death and the circumstances at the time of death may include distressing factors that give rise to an extra risk of long-term psychological morbidity. One such possible distressing factor that may lead to an avoidable psychological trauma of the surviving partner is the patient's suffering (physical or psychological) before death. Cameron and Parkes (1983) reported that adequate pain relief of dying patients may contribute to better adjustment of the bereaved.

Portenoy and Lesage (1999) assess that 70–90% of cancer patients with advanced disease have chronic pain but that it is possible that 90% of them could obtain adequate relief. However, in practice, symptom relief during a cancer patient's last phase of life is not always that efficient (Addington-Hall and McCarthy, 1995). Some reasons for inadequate symptom control may be: insufficient accessibility (distance to health care facilities), a lack of resources (or knowledge) in health care facilities, an emphasis on controlling the disease but not the symptoms or a lack of attention to certain distressing symptoms (e.g. psychological).

Health care should promote well-being, and it is important to identify if insufficient practice enhances avoidable health problems among those losing a partner from cancer. In a previous investigation (Rådestad *et al*, 1996) we found that sub-optimal acts of care during stillbirth resulted in additional psychological traumata – increasing the risk of psychological morbidity among the mothers 3 years after the event. With similar methods as in our previous investigations (Helgason *et al*, 1996; Rådestad *et al*, 1996; Bergmark *et al*, 1999) we investigated if a cancer patient's unrelieved symptoms (physical pain and psychological symptoms) during the last 3 months of life increased the risk of long-term psychological complications in the surviving widow. We utilised the excellent conditions in Sweden, with a unique civil registration number for all citizens and nearly 100% complete population registers, to collect information from a large unselected population of widows who lost their male partner owing to cancer three years earlier.

## MATERIALS AND METHODS

We identified in the Swedish National Register of Causes of Death 928 men who had died in Sweden of prostate cancer in 1996, and of urinary bladder cancer in 1995 and 1996. As the questions concerning the patient's unrelieved symptoms were asked in a time perspective of 3 months before the patient's death, a diagnosis of cancer at least 90 days prior to the date of death was required. The female partners/wives (for simplicity widows) were identified in the Swedish Population Register as having had the same address as the deceased patient at the time of death. Of the 633 women living with the patients at the time of death, 601 widows were alive and living in Sweden on the 1st of April 1999. Of these, 35 had participated in a pilot study, and of the remaining 566, we attempted to enrol the 506 widows, who were under 80 years of age, and had a listed telephone number. To establish comparable population norms for our dependent variables, we randomly chose one married woman (or a woman living with a man) for every two widows, matched for age and region of residence, from the Swedish Population Register. Exclusion was based on the same criteria as in the widowed population, leaving 287 women in the married population.

The widows received an introductory letter explaining the objectives of the study and were then telephoned and asked if they would participate. Those who agreed were sent a questionnaire asking about medical care, the patient's illness and death, and the widow's current life situation and well-being. In order to safeguard the women's anonymity, they sent in the questionnaire separately from an answer card. The study was approved by the Regional Ethics Committee of the Karolinska Institute.

The questions were developed on the basis of successive in-depth interviews and tested with face validity. The face validity procedure was arranged so that the population of widows could answer the questions appropriately, and that there was a consensus of understanding and subjective interpretation of the questions among the widows. The widow's perception of the patient's distress was measured with the following formulations: ‘Did you observe if pain/anxiety/depression affected your partner's well-being during his last 3 months of life?,’ to which there were four possible answers: ‘No, not at all’, ‘Yes, a little’, ‘Yes, moderately’, and ‘Yes, a lot’. The questions concerning the patient's access to pain control had the following multiple choice answers: ‘My partner did not have any need for pain control’, ‘No, access’, ‘Little access’, ‘Moderate access’, and ‘Much access’. Access to psychological support was asked about in a similar manner. The widows' (and the married women's) anxiety, depression, physical health, psychological well-being and quality of life were measured on a seven point visual digital scale using the following formulations: ‘Have you been anxious/depressed during the past week?’ (ranging from ‘Never’ to ‘All the time’), ‘How would you rate your current physical health/psychological well-being/quality of life? (ranging from ‘very bad’ to ‘very good’ or ‘worst possible quality of life’ to ‘best possible quality of life’. The respondents were asked about sleep disturbances and intake of sleeping pills and tranquillisers during the previous month and the possible answers were: ‘No, never’, ‘Yes, once or twice’, ‘Yes, a few times (1–2 times per week)’, ‘Yes, several times (about 3–4 times per week)’ or ‘Yes, nearly always or every day/night’. Two summarising scales were used: Spielberger's Trait measure from the State–Trait Anxiety Inventory (Spielberger *et al*, 1983), and the Centre for Epidemiological Studies (CES-D) measure of depression (Radloff, 1994).

The responses to the questionnaire have been dichotomised, and the results are presented as relative risks; calculated as the proportion of widows with a particular problem who reported high levels of unrelieved symptoms of the deceased divided by the proportion of widows reporting low levels of unrelieved symptoms. Cut-off points correspond to our prior work (Helgason *et al*, 1996; Rådestad *et al*, 1996; Bergmark *et al*, 1999) and were chosen to meet the criteria of clinical significance.

Using the Mantel–Haenszel method (Rothman and Greenland, 1998), estimated relative risks were adjusted for possible confounding factors that have been identified as risk factors of psychological morbidity. These comprise: the women's age (Ball, 1977); marital status and educational level (Rådestad *et al*, 1996); emotional relations/support (Kurz *et al*, 1997); the women's participation in the care of her partner (Häggmark *et al*, 1991); intensity of religious faith; treatment for mental health problems prior to the disease/loss; the site and duration of the patient's illness; and the duration of time the woman was aware of her partner's impending death (awareness time) (identified in this data set).

## RESULTS

Information was supplied by 379 (75%) of the 506 widows. On the average, 3 years had elapsed between the death of the patient and the point in time of the follow-up. The follow-up time (the time since the patient died being 2–3 years compared with 3–4 years) was not associated with reports of the patient's unrelieved symptoms – (RR=1.0 (0.9–1.2) for patient's depression, RR=0.9 (0.8–1.1) for patient's anxiety, RR=1.1 (1.0–1.2) for patient's pain). Table 1

**Table 1:**
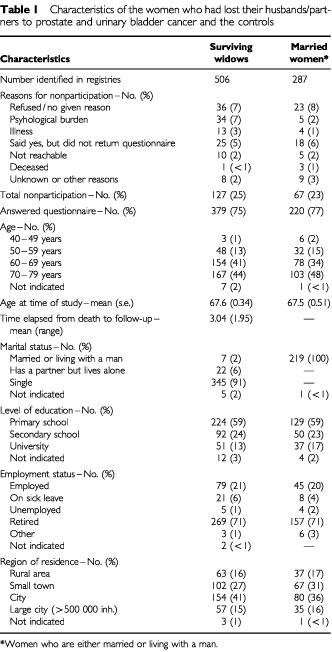
Characteristics of the women who had lost their husbands/partners to prostate and urinary bladder cancer and the controls

shows the widows' (and the married women's) age distribution, marital status, residence region, level of education and employment status.

The patient's mental health status during the last 3 months of life was predictive of the widows' anxiety and depression levels as well as sleep disturbances at the time of follow-up (Tables 2

**Table 2:**
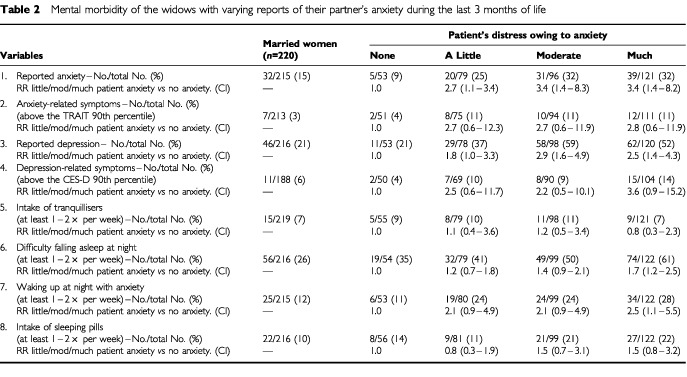
Mental morbidity of the widows with varying reports of their partner's anxiety during the last 3 months of life

and 3

**Table 3:**
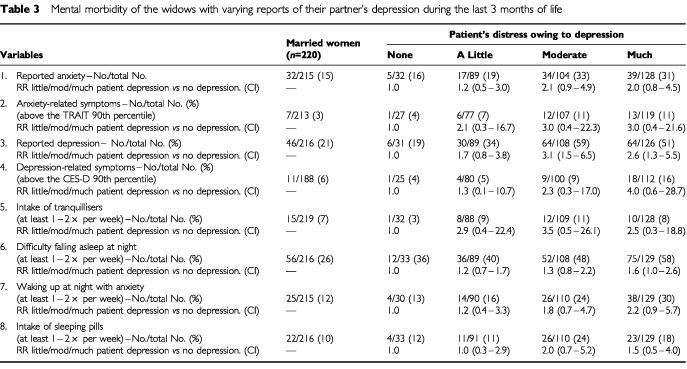
Mental morbidity of the widows with varying reports of their partner's depression during the last 3 months of life

). When the patient was perceived as being very anxious (compared to no observed symptoms) the relative risks for the widow's psychological symptoms were (adjusted for awareness time): 3.6 (1.5–9.4) for anxiety and 2.6 (1.5–4.4) for depression (3-7/7 on the visual digital scales), 3.1 (0.7–13.0) for anxiety related symptoms (above the 90th percentile on STAI-T), 3.4 (0.9–13.6) for depression related symptoms (above the 90th percentile on CES-D), 1.8 (1.2–2.6) for difficulty in falling asleep at night and 2.5 (1.1–5.5) for waking up at night with anxiety. Similar increases in the widow's risk of psychological complications were obtained when the patient was considered to have been very depressed during his last months of life.

Reports of the patient's distress owing to pain were not associated with the widow's mental health at follow-up (Table 4

**Table 4:**
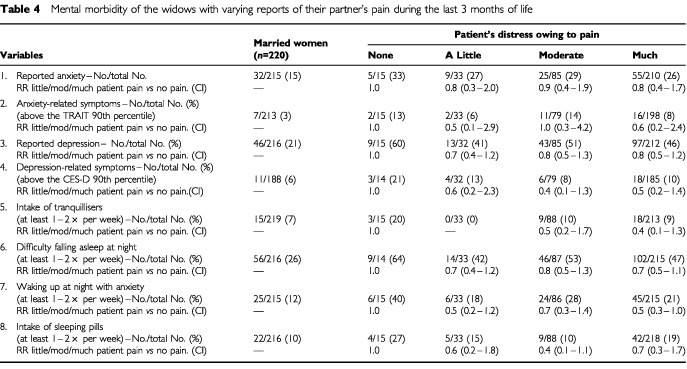
Mental morbidity of the widows with varying reports of their partner's pain during the last 3 months of life

). When the patient was in much pain (compared to no observed symptoms) the relative risks for widows' psychological complications were (adjusted for awareness time): 0.9 (0.4–1.9) for anxiety and 0.8 (0.5–1.3) for depression (3-7/7 on the visual digital scales).

We examined additional variables, and found, just as for pain, no relation to the widow's mental health at follow-up (not in table). The relative risks for the widows' depression (visual digital scale) were: 1.0 (0.8–1.1) for when the patients had had much symptoms in the urinary tract (compared to no observed symptoms), 1.0 (0.7–1.5) for oedema, 1.0 (0.8–1.1) for constipation, 1.0 (0.7–1.4) for vomiting, and 1.1 (0.8–1.4) for disrupted sleep owing to urinary tract symptoms.

Sixty-six per cent (242 out of 366) of the patients were assessed to have been moderately or much depressed during the last 3 months of life, 62% (222 out of 360) as anxious and 87% (309 out of 357) in pain. A considerable difference was noted in access to pain control and psychological support. While a small minority of the women stated that their husbands had not needed the services; of the remaining majority 93% had moderate or much access to pain control during the last 3 months of life compared to only 33% regarding psychological support (Table 5

**Table 5:**
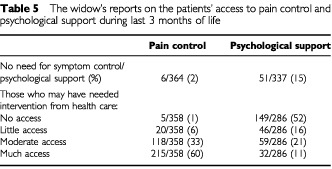
The widow's reports on the patients' access to pain control and psychological support during last 3 months of life

).

Stratum specific and adjusted relative risks were calculated by the Mantel–Haenszel method (data not shown). The relationship between the patient's anxiety and the widow's depression is slightly weakened when controlled for treatment for mental health problems before the patient's illness; however, this group of treated women is very small and the relationship is intact for the others. The relative risks increase somewhat after adjusting for the widow's awareness time, i.e. the time the woman was aware of her partner's impending death. Marital status shows tendency to modify (not significantly) – the relationship is hardly apparent among widows who have remarried or have a subsequent relationship.

## DISCUSSION

Inefficient psychological symptom control during the last months of a cancer patient's life may predispose the surviving partner to long-term psychological morbidity. The widows assessed that the patients had good access to pain control, but poor access to psychological support.

To our knowledge, our study is the first investigating the relationship between the patient's psychological condition during the last months of life and psychological morbidity in surviving partner 3 years later. This fact, and validity issues inherent in our observational design, hinders a definite conclusion concerning causality. Several previous studies give indirect support for our findings. It has been reported that the well-being of cancer patients and their family caregivers is interrelated during the illness (Cassileth *et al*, 1985; Kurz *et al*, 1995; Kristjanson *et al*, 1996; Hodgson *et al*, 1997) and others have found the mental condition of the well partner during the terminal period to be the main risk factor of morbidity in widowhood (Kelly *et al*, 1999). We have no indication of that validity problems explain our findings. One evident validity concern is if women with psychological complications have a general tendency to over-report unrelieved symptoms of their husband. We found that the patient's unrelieved pain, symptoms of the urinary tract, oedema, constipation, vomiting and disrupted sleep owing to urinary tract symptoms, gave relative risks for the widows anxiety and depression close to 1.0, indicating that a general over-reporting cannot explain our findings (further validity discussion is given below). The differences in access to physical pain relief and psychological symptom relief (support/drugs) offers a probable explanation for the differential effects on the women's psychological morbidity.

A mechanism explaining the relationship between the patient's psychological symptoms and the widow's depression 3 years later may be ‘learned helplessness’. The woman was not able to help her partner/husband with his anxiety or depression, nor did the health care professionals ameliorate the symptoms. She was thus exposed to chronic distressing stimuli from which she could not escape – a situation that may give rise to a feeling of helplessness or a hopeless expectation towards life. Exposure to inescapable, distressing stimuli has been associated with a response of helplessness in animals – a behaviour that resembles human depression (Seligman, 1974). Using a role playing technique (‘empty chair method’), 73 bereaved spouses (for 6 months) were given a last opportunity to talk with their deceased spouse. Levels of unresolved grief during the role playing session (e.g., anger, guilt, helplessness, non-acceptance) predicted levels of depression (Beck's Depression Inventory) and subjective distress (Impact of Event Scale) 8 months later (Field and Horowitz, 1998). Moreover, uncontrollable events predicted higher depression levels among caregiving spouses of Alzheimer patients (Pagel *et al*, 1985), possibly mediated though the mechanism of learned helplessness.

Intrusive, painful memories of the patient's mental suffering and avoidance of stimuli that trigger these, may explain the increased percentage of women reporting anxiety and anxiety related symptoms (e.g., STAI-T and sleep disturbances). It is possible that our documentation of the increased risk of anxiety after the exposure to patient's unrelieved symptoms may partly resemble a Post-traumatic Stress Disorder. The condition has previously been reported after a loss of a partner of chronic illness (Zisook *et al*, 1998).

Contrary to expectations, the widow's long-term psychological morbidity was not associated with the widow's evaluation of the degree of pain experienced by her late partner. It is possible that our question of somatic pain was too crude, and that a more sensitive measure is needed to investigate the relationship. It is also possible that the woman did not feel responsible for relieving the patient's physical pain and therefore did not acquire a feeling of helplessness or guilt leading to subsequent mental health symptoms. Furthermore, access to pain relief was satisfactory, so, even if the patient experienced pain, efforts were made to relieve it so the situation may not have been perceived as ‘uncontrollable’ at all. Moreover, the woman may have been able to make an effort – call for help, or deliver morphine as prescribed.

The widows' reports clearly illustrate current possibilities in Sweden to improve the mental health condition of terminally ill cancer patients. About two thirds of the patients are judged to have been moderately or highly anxious/depressed during their last 3 months of life, while only 29% of these had satisfactory access to psychological support and only about 50% of them were given psychoactive drugs. Previous research from England (Fallowfield *et al*, 2001) and Austria (Sollner *et al*, 2001) indicates that physicians caring for cancer patients may be poor at identifying psychological symptoms among their patients.

Our study is observational, and thus has a large number of validity concerns that have been addressed in the design, execution and analysis (Steineck *et al*, 1998). Our population based registers, from which we were able to retrieve a non-selected nationwide cohort of widows, diminish problems pertaining to selection and give us full control over the denominator in calculating the participation rate. In order to disentangle the effect of possible predictors from other aspects related to the specific disease site, we decided to have as homogenous a group as possible and only include women who's husband/partner had died owing to these two cancer sites. However, we needed these two kinds of cancer sites in order to get a large enough number for the statistical analysis. Based on our previous investigations, we expected the response rate to be low for women 80 years and older. We excluded them from the study, both for their sake (too much work load) and for the sake of validity. The follow-up time interval was a compromise between the objective of investigating long-term morbidity and the women's possible memory capacity. In preparatory interviews, we studied the time interval that could possibly elapse between the loss and follow-up, in order for the women to be able to recall the event and answer the questions. Furthermore, additional analysis of our material revealed that the follow-up time (being 2–3 years or 3–4 years) did not matter for the widows' evaluation of the patients' unrelieved symptoms. The questionnaire was designed to collect information on a number of potential confounders. The association between the patient's anxiety and the widow's risk of depression was weaker among women treated for mental health problems prior to the partner's illness, compared to other women. The relative risks change somewhat after adjustment for this variable but the subgroup is too small and the relationship is intact for widows that have not been treated for mental health problems. The relative risks change little, if at all, on adjusting for the remaining potential confounding factors, indicating that confounding owing to these factors cannot explain our findings.

We have used established scales for measuring anxiety related symptoms (STAI-T) and depression related symptoms (CES-D), and added clear direct questions (tested by face validity and previous investigations) (Helgason *et al*, 1996; Rådestad *et al*, 1996; Bergmark *et al*, 1999) pertaining to anxiety, depression, and consumption of tranquillising drugs. Our questions about the patient's condition were tested by face validity and in preparatory studies. Furthermore, in the analysis of our data we contemplated whether women with psychological complications had a general tendency to over-report the unrelieved symptoms of their husbands/partners. We found no indication of such differential reporting to explain our findings.

To sum up, our findings imply that unrelieved psychological symptoms of terminally ill cancer patient's increase the risk of long-term psychological morbidity of their surviving widows. Efficiency in diagnosing and treating psychological complications of terminally ill cancer patient's may not only improve their quality of life but possibly also prevent long-term psychological morbidity of their surviving partners.
